# Human punishment is motivated by inequity aversion, not a desire for reciprocity

**DOI:** 10.1098/rsbl.2012.0470

**Published:** 2012-07-18

**Authors:** N. J. Raihani, K. McAuliffe

**Affiliations:** 1 Department of Genetics Evolution and Environment, University College London, London WC1E 6BT, UK; 2 Department of Human Evolutionary Biology, Harvard University, Cambridge, MA 02138, USA

**Keywords:** punishment, inequity aversion, reciprocity

## Abstract

Humans involved in cooperative interactions willingly pay a cost to punish cheats. However, the proximate motives underpinning punitive behaviour are currently debated. Individuals who interact with cheats experience losses, but they also experience lower payoffs than the cheating partner. Thus, the negative emotions that trigger punishment may stem from a desire to reciprocate losses or from inequity aversion. Previous studies have not disentangled these possibilities. Here, we use an experimental approach to ask whether punishment is motivated by inequity aversion or by a desire for reciprocity. We show that humans punish cheats only when cheating produces disadvantageous inequity, while there is no evidence for reciprocity. This finding challenges the notion that punishment is motivated by a simple desire to reciprocally harm cheats and shows that victims compare their own payoffs with those of partners when making punishment decisions.

## 1. Introduction

Punishment is a costly behaviour that is often aimed at individuals who cheat during social interactions. Although punishers make an initial investment to harm cheats, the investment may be repaid if the cheat behaves more cooperatively in future interactions [1,2]. Identifying the motives underpinning human punishment is crucial as punishment plays an important role in the maintenance of cooperation in human societies [3]. Several recent studies have shown that players experience negative emotions, such as anger or disgust, when they interact with cheats and that the intensity of these emotions is positively associated with the desire to reciprocally harm cheating partners [4–6]. The act of administering punishment provides relief from negative emotions as it activates reward centres in the brain [7]. In this way, punishment can be subjectively rewarding. Although negative emotions motivate punishment, it is not yet clear why these emotions are produced during interactions with cheats. One possibility is that negative emotions are caused by disadvantageous inequity aversion (hereafter ‘inequity aversion’), or the disutility associated with experiencing lower payoffs than a cheating partner [8]. However, a simpler alternative is that victims of cheats experience negative emotions because cheats violate cooperative norms, thereby imposing losses on cooperative partners [4]. Thus, in some contexts, punishment may be motivated by the desire to reciprocally harm cheating partners, even if the cheating partner did not experience higher payoffs than the victim. Thus, experiencing losses without simultaneously experiencing unequal payoffs may suffice to motivate punishment, although this possibility has not been tested.

The concepts of inequity aversion and of reciprocity both predict that punishment decisions will be influenced by the partner's behaviour. However, inequity aversion predicts that individuals are sensitive to how their payoffs compare with those of interaction partners, whereas the concept of reciprocity predicts that punishment decisions are influenced by how payoffs compare with individual expectations and are therefore independent of the relative payoffs gained by cheating partners. It is hard to disentangle whether punishment is motivated by a desire to reciprocate losses or by inequity aversion because players involved in interactions with cheats often simultaneously experience losses and inequity [9,10]. For example, evidence from laboratory public goods games (where contributions to a communal account are altruistic in the sense that they yield benefits to other group members at a cost to the donor; [11]) has shown that players experience anger and disgust when interacting with non-contributing (‘free-riding’) group members. The intensity of these emotions correlates with the propensity to administer costly punishment to reduce free-riders' incomes [4,12]. However, in such games, cooperative individuals (those who contribute to the communal account) experience absolute losses and lower payoffs relative to those of free-riders, meaning that it is not clear whether the negative emotions produced from interactions with cheats arise from a desire to reciprocate losses or from inequity aversion [10]. A more recent study used a random income game (where players were randomly allocated different-sized earnings) to show that punitive behaviour can be motivated by inequity, even in the absence of losses [12]. Low-earning players in this game experienced negative emotions targeted towards higher-earning counterparts and were willing to pay a cost to reduce the income of high earners [12]. Thus, punitive behaviour can arise even when higher-earning players do not impose losses on lower-earning individuals and when there is no cooperative norm to be enforced. In a subsequent study, it was shown that the tendency to reduce the income of high earners in a random income game is positively associated with the propensity to punish free-riders in public goods games [13]. While these findings indicate that punishment may be motivated by inequity aversion, neither study asked whether punishment might also be motivated by losses in the absence of inequity. We tested this possibility here.

We designed an experiment based on a simplified version of the moonlighting game [14] to determine whether human punishment is motivated by a desire to reciprocate losses or by inequity aversion. Subjects were assigned to one of two roles, player 1 (P1) or player 2 (P2), and were allocated money according to one of three treatments (A–C). In treatment A, P1 was given $0.70 and P2 was given $0.10. In treatment B, P1 was given $0.70 and P2 was given $0.30. In treatment C, P1 was given $0.70 and P2 was given $0.70. The game consisted of two stages: in the first stage, P2 could choose to ‘cheat’ by taking $0.20 of P1's endowment and in the second stage P1 could choose to punish P2 (pay $0.10 to reduce P2's income by $0.30). In treatment A, P1 maintained a higher payoff than P2 when P2 cheated ($0.50 versus $0.30). In treatment B, cheating by P2 produced equal outcomes ($0.50 each). In treatment C, cheating by P2 meant that P1 got $0.50 while P2 got $0.90. Thus, P1 experienced the same losses in all three treatments and always finished with a payoff of $0.50 if P2 cheated. Crucially, however, only in treatment C did cheating by P2 result in P1 experiencing lower payoffs than P2 (disadvantageous inequity). This setup therefore allowed us to disentangle the effect of experiencing losses from the effect of experiencing inequity as motivators for punishment.

## 2. Material and methods

We recruited subjects for our experiment using the online labour market, Amazon Mechanical Turk (AMT; www.mturk.com). AMT connects ‘requesters’ (or experimenters) with ‘workers’ (or subjects), the latter being incentivized to perform short tasks for small payments [15]. Previous studies have validated this approach by replicating findings from economic games performed under laboratory conditions [16–18], even with small stakes that are commonly used in the AMT framework [19].

We recruited 560 subjects (361 males, 195 females, four unspecified) to take part in our experiment. Subjects varied in age from 16 to 69 (mean = 29 ± 0.4) years old. Of the 560 subjects, 280 (175 males, 101 females, four unspecified) were allocated the role of P1 and assigned to one of six independent treatments (see the electronic supplementary material, tables S1 and S2). The remaining 280 subjects were assigned the role P2 and were assigned to one of three treatments (see the electronic supplementary material, table S2). Each subject was allocated to one role and one treatment only. We used ex-post matching [18] to pair players with their respective partners. All subjects were paid a show-up fee of $0.20 and were required to answer four comprehension questions correctly to take part in the experiment. Eligible subjects were redirected to an external survey website (https://opinio.ucl.ac.uk) where they were assigned to their role (P1 or P2) and treatment. Subjects played anonymously because they interact via a unique, 14-digit worker ID and were told that their worker ID would not be revealed to other players in the game. Workers were prevented from participating repeatedly in the experiment by allowing only one entry per unique ID (worker ID must be linked to a valid credit card, which largely prevents workers from accruing multiple accounts; [16]) and by preventing repeated access to the external survey website from the same IP address.

## 3. Results

**Figure 1.**
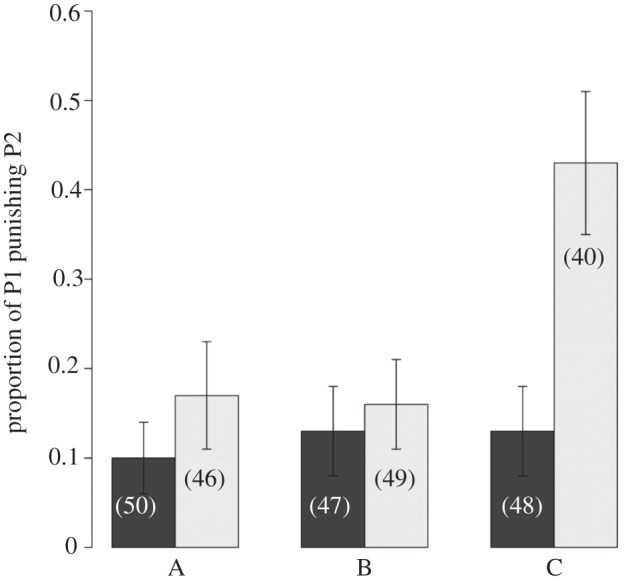
The proportion of P1 individuals who punished P2 according to treatment (A–C) and whether P2 cheated (by taking $0.20 of P1's endowment). Initial endowments (P1: P2, $) in treatment A were 0.70 : 0.10; in treatment B were 0.70 : 0.30 and in treatment C were 0.70 : 0.70. Sample sizes for each condition are indicated in parentheses. Dark grey bars, no cheating; light grey bars, P2 cheated.

The mean proportion of P1 who decided to punish P2 was 24.4 ± 0 per cent when P2 cheated and 11.7 ± 0 per cent when P2 did not cheat. In treatments A and B, P1's decision to punish P2 was not affected by whether P2 cheated (figure 1). Thus, incurring losses in the absence of inequity did not motivate P1 to punish P2. Conversely, experiencing losses that also produced inequity (treatment C) motivated P1 to punish P2 (χ^2^ = 10, *p* = 0.007, figure 1).

## 4. Discussion

In this experiment, the loss experienced by P1 as a result of P2 cheating was the same ($0.20) across all treatments but P1 only punished P2 when P2 cheating resulted in P1 experiencing lower relative payoffs. Together, these results suggest that disadvantageous inequity is the driving force motivating punishment, implying that the proximate motives underpinning human punishment might therefore stem from inequity aversion rather than the desire to reciprocate losses. Although evidence for punishment in non-human animals is rare [2], in species where it does occur it is thought to be motivated by experiencing losses rather than inequity [10]. In part, this may be because monitoring their own payoffs relative to an internal reference point is less cognitively demanding than also monitoring—and comparing—the payoffs that accrue to an interaction partner.

Why do humans rely on a more cognitively complex task of monitoring their own payoffs relative to those of interaction partners, rather than simply monitoring their own payoffs relative to an internal expectation, when deciding whether to punish cheats? One possibility is that punishment promotes cooperative behaviour only if the punishment is deemed to be fair. This may only be the case when a cheat gains higher payoffs from the interaction than the victim as a consequence of the cheating behaviour. Victims of cheats may therefore do best to monitor how their payoffs compare with those of interaction partners before investing in costly punishment. Empirical evidence offers some support for this hypothesis. Using an experimental trust game with and without punishment, Fehr & Rockenbach [20] showed that the moral legitimacy of punishment has a striking effect on the prevalence of cooperative behaviour. In this game, one individual, an ‘investor’, can send money to another individual, a ‘trustee’. The money sent to the trustee is multiplied by the experimenter and the trustee can then choose how much of the entrusted amount to return to the investor. In their study [20], investors were allowed to stipulate how much of their initial investment they expected to receive back from the trustee and also whether they would fine the trustee for non-compliance. They found that fines for desired back-transfers that were deemed to be unfair largely undermined cooperative behaviour: trustees kept more of the invested amount under these conditions. This study suggests that punitive behaviour that is deemed to be unfair may not be accepted by the target. As well as failing to promote cooperative behaviour, punishment may also elicit retaliation from targets (cf. [21]), although it is not known whether retaliation is more likely when punishment is deemed to be unfair. This would be an interesting avenue for future investigation.

To sum up, our experiment demonstrates that humans are sensitive to inequity but not to losses when deciding whether to punish a cheating partner. Elucidating the motives that trigger punishment of cheats may yield insights into the ultimate function of punitive behaviour in humans: specifically, it may be the case that punishment is aimed at promoting fair behaviour rather than simply deterring partners from cheating. Such insights may tell us much about the contexts where punishment is most likely to be implemented and also where it is likely to be most effective.
